# Acceptability of Human Papillomavirus Self-Sampling for Cervical Cancer Screening in an Indigenous Community in Guatemala

**DOI:** 10.1200/JGO.2016.005629

**Published:** 2017-01-18

**Authors:** Anna Gottschlich, Alvaro Rivera-Andrade, Edwin Grajeda, Christian Alvarez, Carlos Mendoza Montano, Rafael Meza

**Affiliations:** **Anna Gottschlich**, **Christian Alvarez**, and **Rafael Meza**, University of Michigan, Ann Arbor, MI; **Alvaro Rivera-Andrade** and **Carlos Mendoza Montano**, Institute of Nutrition of Central America and Panama; **Carlos Mendoza Montano**, Universidad Mariano Galvez de Guatemala; **Edwin Grajeda**, Universidad Rafael Landivar, Guatemala City, Guatemala; and **Alvaro Rivera-Andrade**, The Hebrew University, Jerusalem, Israel.

## Abstract

**Purpose:**

Cervical cancer rates in Latin America are higher than those in developed countries, likely because of the lower prevalence of screening. Specifically, less than 40% of women in Guatemala are regularly screened and even fewer women are screened in indigenous communities. Current screening strategies—Pap smears and visual inspection with acetic acid—might not be the most effective methods for controlling cancer in these settings. We thus investigated the potential of self-collection of cervical samples with testing for human papillomavirus (HPV) to help prevent cervical cancer in an indigenous community in Guatemala.

**Patients and Methods:**

A community representative random sample of 202 indigenous women age 18 to 60 years residing in Santiago Atitlan, Guatemala, were surveyed to assess knowledge of and risk factors for HPV and cervical cancer. Women were then invited to self-collect a cervical sample using HerSwab collection kits to assess the prevalence of HPV and the acceptability of self-sampling.

**Results:**

Of 202 women who completed the survey, 178 (89%) provided a self-sample. In all, 79% of these women found the test comfortable, 91% found the test easy to use, and 100% reported they were willing to perform the test periodically as a screening method. Thirty-one samples (17%) were positive for at least one of 13 high-risk HPV types, and eight (4.5%) were positive for HPV 16/18.

**Conclusion:**

HPV testing by using self-collected samples was well accepted, suggesting that it is a plausible modality for cervical cancer screening in indigenous communities. Further studies are needed to assess rates of follow-up after a positive test and to determine whether these findings extend to other indigenous and nonindigenous communities in Guatemala and Latin America.

## INTRODUCTION

Cervical cancer (CC) is preventable with appropriate screening and treatment. Pap smears, the most common form of screening, allow physicians to detect and manage pre-cancerous lesions before they develop into CC.^1^

Because of the success of screening programs that use the Pap smear, CC rates are low in most high-income countries.^2,3^ Nonetheless, CC is the third most common cancer worldwide and a leading cause of death among women in low- and middle-income countries (LMICs).^4^ Unfortunately, Pap smears are infrequently used in LMICs because they are expensive and require physicians, pathologists, and cytotechnicians to perform the procedure and interpret the results.^3,5^ Even in LMICs with screening programs, rates of participation tend to be low^6^ because Pap smears must be collected and analyzed at hospitals or other high-resource health facilities that women may not have access to.

In addition, if women have abnormal results, they must return for follow-up assessment and/or treatment, which creates greater time and financial burdens.^7^ The logistics of sample collection by health care providers, which then must be sent to laboratories, tested, and returned, can also be challenging in these settings. There are also cultural barriers that preclude the use of screening methods associated with sexually transmitted diseases (STDs).

Hence, many LMICs have adopted CC screening programs that use visual inspection with acetic acid (VIA). VIA involves placing acetic acid on the cervix and looking for a change in color to detect lesions. This procedure is less costly and invasive than Pap smears and can be performed by trained laypersons in low-resource health facilities.^7-9^ In addition, VIAs give the option to treat women with cervical lesions immediately. Thus VIA is often called a “see/screen-and-treat” or “one-visit” approach.^7^ Previous studies have shown that VIA screening helps reduce CC incidence and mortality in low-resource settings.^8^ However, VIA shares some of the same barriers associated with Pap smears, so despite these efforts, CC incidence and mortality remain high in many LMICs, presumably because of persistent low rates of screening with either approach.

Human papillomavirus (HPV) infections are responsible for more than 90% of CC cases.^10,11^ There are 13 types of high-risk HPV associated with development of CC.^12^ Of these, types 16 and 18 account for approximately 70% of all cases.^13^ Cervical HPV tests have high sensitivity (approximately 90%) and specificity (> 80%).^14,15^ Women who test positive for high-risk HPV should follow up with a Pap smear and/or VIA or treatment, depending on each country’s setting and resources,^16^ but a negative test means the risk of developing CC in the next few years is minimal, lower than the risk after a negative Pap smear.^17^ Furthermore, when Pap smears are performed only on women who have tested positive for HPV, the relatively low sensitivity of screening by using Pap smears is significantly improved.^15,18^ Thus, primary screening for high-risk HPV before referral for Pap smear or VIA has been proposed as an alternative CC screening method. Unfortunately, HPV testing is expensive and requires infrastructure not readily available in many LMICs. Nonetheless, research is underway to develop low-cost HPV tests that can be used with minor infrastructure requirements.^19-23^

Self-collection HPV kits have been developed to allow women to collect their own cervicovaginal samples at home and send these to a testing facility through the mail or by other means. Studies in several countries have compared the accuracy of HPV self-collection samples with samples obtained by a physician and have assessed the acceptability of self-collection in different populations.^5,24-30^ Some studies have provided women with self-collection kits, but at medical facilities before collection by a physician rather than at the woman’s home. In these studies, self-collection has been shown to have sensitivity similar to that of physician-collected samples,^5,24-28^ and self-collection has been found to be highly acceptable in many settings.^24,26-28,31^ This suggests that self-collection could be helpful to increase CC screening rates in LMICs, once cost- and infrastructure-efficient HPV tests have been developed. However, few studies have provided participants with the opportunity to try these in community settings outside of medical facilities; thus, it is not clear whether they would be an accepted form of primary CC screening.

Guatemala has one of the highest levels of CC morbidity and mortality in the region. Age-standardized annual incidence and mortality rates are 22.3 and 12.5 per 100,000 women, respectively,^11^ largely because less than 40% of Guatemalan women (who have a relatively high prevalence of HPV^32^^-34^) have ever been screened for CC.^6,35^ There have been self-collection studies conducted in Latin America, a region in which CC morbidity and mortality are particularly high,^5,36-39^ although few have tested the acceptability of HPV self-collection in community rather than clinical settings. Moreover, HPV self-collection has not been studied in indigenous populations in Latin America, who tend to have less access to health facilities and higher levels of stigma associated with physician-administered vaginal and STD tests.^40^ Thus, it is important to assess the acceptability of HPV self-collection kits and tests and determine the potential of HPV testing as a screening modality in these settings.^37^ We thus conducted a cross-sectional study in an indigenous population in Lake Atitlan, Guatemala, to assess knowledge of HPV and CC, provide women with the opportunity to collect a self-sample in their home and report their feelings and experiences, and assess HPV prevalence in indigenous populations.

## PATIENTS AND METHODS

We conducted a cross-sectional study in Santiago Atitlan, an indigenous community of 45,000 residents in Guatemala. Data were collected by using electronic surveys and self-collection kits.

### Study Population

This community is almost exclusively Tz’utujil, a Mayan indigenous group. We sampled 212 women age 18 to 60 years from nine neighborhoods that encompass 85% of the population of Santiago Atitlan. Population data were obtained from the local municipality. We followed a stratified sampling approach by first allocating samples of size *N_c_* to each neighborhood according to its relative population size (*c =* 1,…,9) and then randomly selecting a sample of *N_c_* blocks. One house was randomly selected per block, in which one woman was interviewed.

If more than one woman in a house was eligible, the woman who had the next upcoming birthday was selected. Only women ages 25 to 54 years were eligible to provide a self-collected sample for HPV testing because women outside these ages are not eligible to receive screening using Pap smears or VIA according to Guatemalan CC screening guidelines. Menstruating and pregnant women were also excluded from self-collection. We chose to interview women outside the screening range because, although the focus of the study was on acceptability, we were interested in learning about the health practices and risk factors for all adult women.

### Survey

The survey component was designed by using the Qualtrics application. It included 143 questions about demographics, preventive health care practices, and HPV and CC knowledge and risk factors. The survey also assessed the acceptability of and feelings toward HPV self-collection. Questions were developed by using the STEPwise Approach to Surveillance (STEPS) survey, The University of North Carolina’s Family Health Study Survey, and the University of Michigan’s Michigan HPV and Oropharyngeal Cancer study.^35,41^ Four trained community health workers (CHWs) fluent in Tz’utujil and Spanish conducted the surveys.

The survey was written in English and then translated to Spanish by native speakers from the study team. The survey was piloted in Guatemala City and in households in Santiago Atitlan. After each pilot, surveyor notes were reviewed and appropriate revisions were made. At the end of each day, surveys were uploaded to the server, ensuring that the participant’s data could no longer be accessed, except by members of the study team.

### HPV Self-Collected Samples

We used Eve Medical HerSwab self-collection HPV kits. Each kit came with an instructions card written in Spanish with step-by-step infographs explaining the collection process. The CHWs were trained on the procedure and on how to explain the instructions to the participants in their native language.

Upon interview completion, each eligible participant was asked about her interest in collecting a sample for HPV testing. If the participant agreed, the CHWs explained the instructions, and the participant collected a sample in a private room in the household. The collection kit comprised a plastic handle and brush. The woman inserted the brush into her vagina and then turned a crank on the handle to extend the brush. The woman then removed the brush and cranked it back by using the handle. She then returned the kit to the CHWs. Afterward, each participant completed a five-question survey assessing the level of ease and comfort associated with the collection and her willingness to self-collect periodically as a form of CC screening. Finally, CHWs encouraged participants to attend free VIA screening clinics at their local public hospital.

Samples were sent to an independent, nonprofit laboratory in Guatemala City (Asociación de Salud Integral) for testing and were tested by using the Anyplex II^42^ HPV-28 kit, which tests for 13 high-risk HPV types according to the International Agency for Research on Cancer classification,^12^ as well as 15 low-risk types (Data Supplement).

To ensure the privacy and confidentiality of the participant’s information, given the sensitivity of the survey questions and the HPV test, no contact information was collected in this pilot study; thus, participants could not be contacted by the study team with their results. Instead, participants were told to call for their results 10 days after collection by using an identification number. Announcements were made daily on the local radio for 1 month after the end of recruitment that reminded women to call for their results. Participants were informed only if they tested positive for one of the 13 high-risk types.

### Statistical Analyses

Post–self-collection survey questions were analyzed to determine the acceptability of HPV self-collection as a form of CC screening. Two additional outcomes were analyzed: positive HPV results and previous Pap smear or VIA results. Crude comparisons between these and relevant covariates were run by using log-binomial regression, and then models were run adjusting for other covariates. Statistical analyses were conducted by using SAS software Version 9.4 (SAS Institute, Cary, NC).

### Human Subjects Approval

The University of Michigan Institutional Review Board approved study protocols (HUM00096559). All participants gave oral, informed consent before participation. The consent was documented by signature from one of the CHWs.

## RESULTS

Of 481 women who were asked to participate through door-to-door recruitment, 212 women enrolled (44% acceptance rate), with 202 (95%) completing the survey. Ten women chose to withdraw, and their data were destroyed. Participants’ mean age was 34.5 years, and more than 80% had primary education at most (Table 1). One hundred thirty-five women (67%) reported previous CC screening with Pap smears and/or VIA (Table 1). Women with previous Pap smear and/or VIA testing tended to be older, married, and with a higher number of children and pregnancies, suggesting that access to screening is strongly tied to reproductive care. Whereas only 31 participants (15%) reported previous knowledge of HPV, 188 (93%) were interested in and willing to collect a self-sample for HPV testing (Table 2). Of these, 178 (88%) were eligible and provided a sample.

**Table 1:**
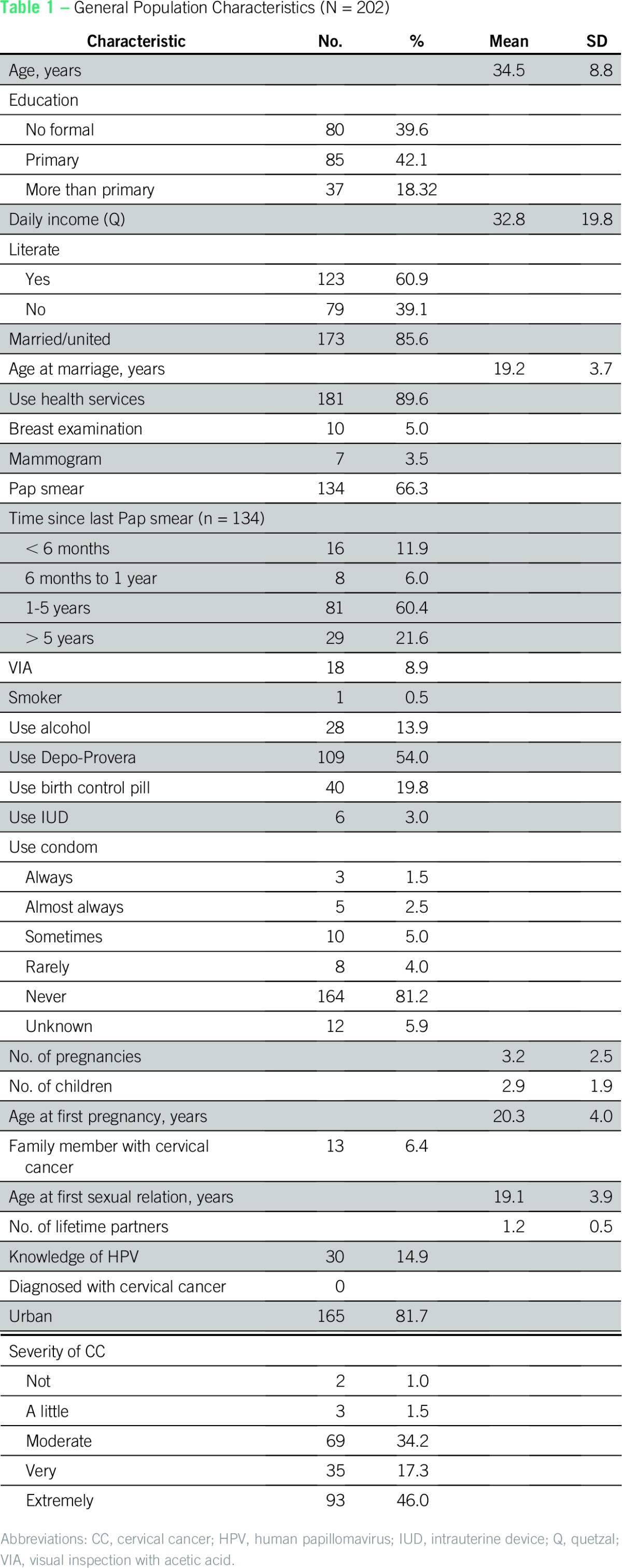
General Population Characteristics (N = 202)

**Table 2:**
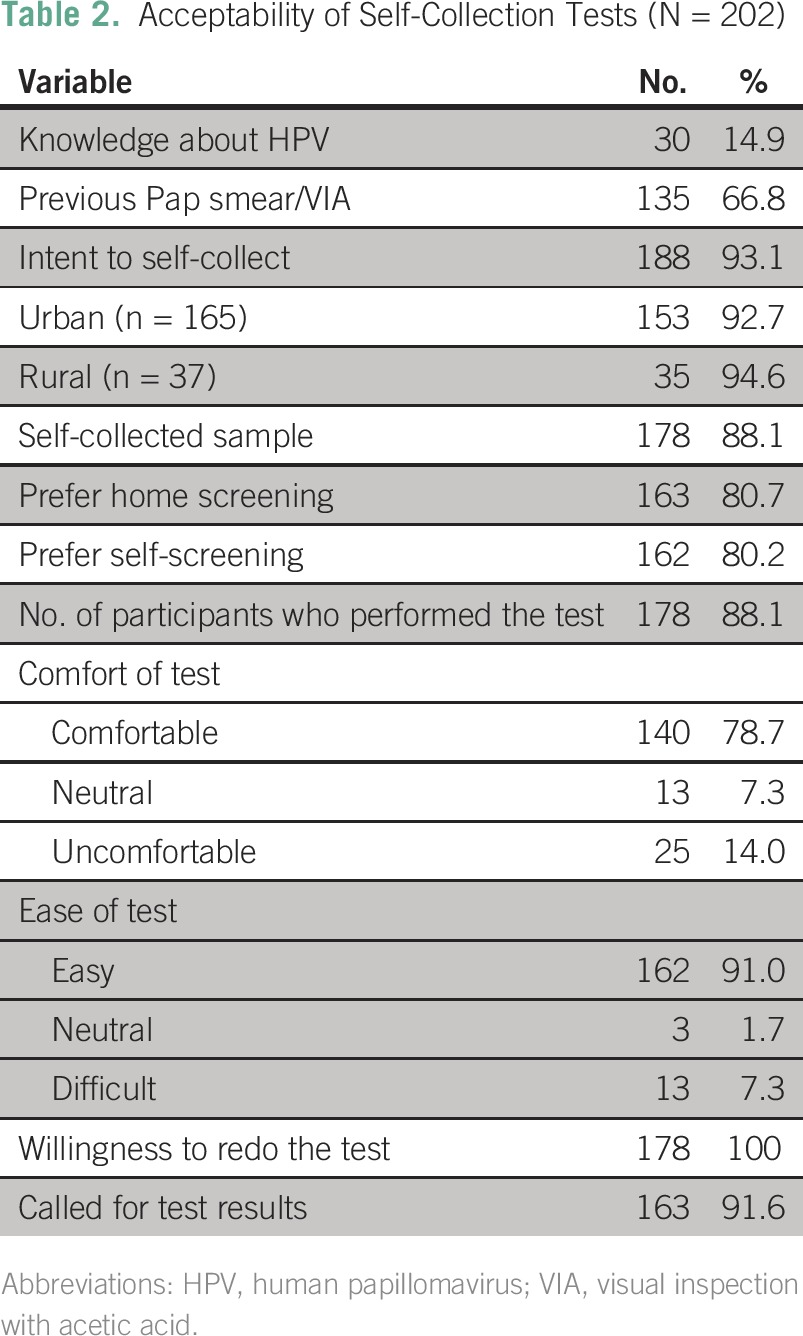
Acceptability of Self-Collection Tests (N = 202)

### Self-Collection Acceptability

Of these 178 women, 79% found the kit comfortable to use, and 91% found it easy to use. Upon collection, 100% reported that they were willing to use the test periodically as a form of CC screening, and more than 80% said they preferred to screen themselves at home rather than with a physician in a doctor’s office (Tables 2 and 3). Because identifying information was not collected, the study team was unable to actively return results; however, more than 90% of participants called to receive their own results.

**Table 3:**
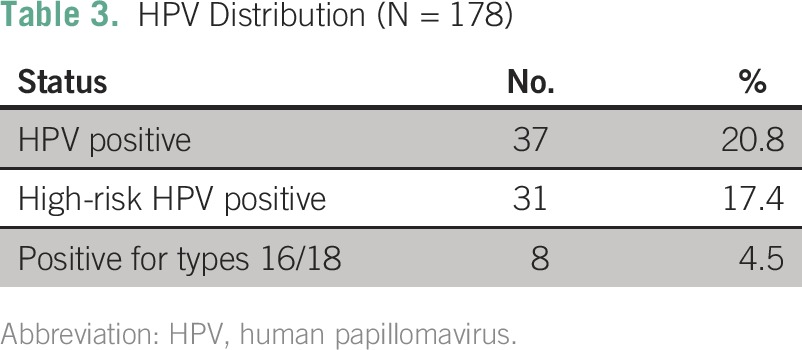
HPV Distribution (N = 178)

### HPV Prevalence

**Fig 1:**
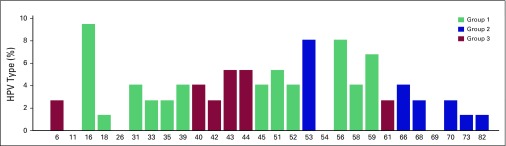
Distribution of human papillomavirus (HPV) genotypes among the 37 HPV-positive samples. Several women had prevalent infections with more than one type; thus, these percentages represent the number of infections from a specific type of HPV divided by the total number of HPV infections. Group 1: most potent HPV type known to cause cancer at several sites or sufficient evidence for cervical cancer; group 2: limited evidence in humans with varying levels of evidence for cervical cancer; group 3: no evidence in humans for cancer.

Thirty-seven (21%) of 178 women tested positive for one of 28 types of HPV, and 31 (17%) tested positive for a high-risk type (Table 3). HPV 16 had the highest prevalence with seven women testing positive, followed by HPV 53 and 56 (six women tested positive for each), and HPV 59 (five women tested positive). Of the four strains with the highest prevalence, all except HPV 53 are high risk. Figure 1 shows the HPV type distribution in the study population.

### HPV Infection

The number of lifetime sexual partners was significantly higher in women who tested positive for HPV. Characteristics comparing women by HPV test results can be found in Table 4; characteristics comparing women by their number of sexual partners can be found in the Data Supplement. Exposure covariates in the final model include current age, level of education, age at first pregnancy, and age at first sexual encounter. Other covariates were also explored, including age at marriage and other demographic factors.

**Table 4:**
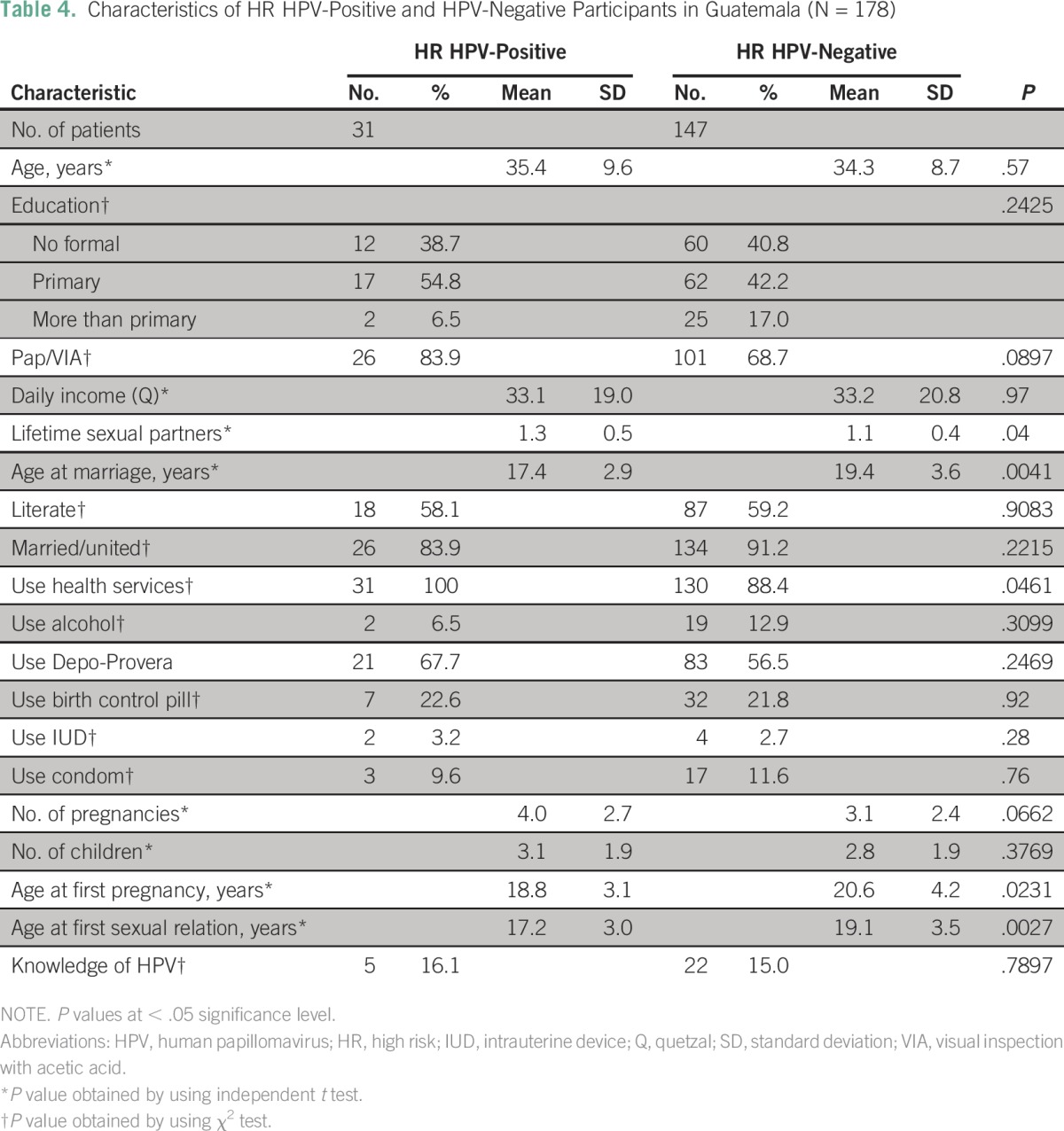
Characteristics of HR HPV-Positive and HPV-Negative Participants in Guatemala (N = 178)

After adjustment, the association became statistically nonsignificant, but it did show a prevalence ratio (PR) greater than 1 (crude PR, 2.18; 95% CI, 1.07 to 4.43; *P* = .03; adjusted PR, 1.42; 95% CI, 0.68 to 2.97; *P* = .34; regression tables are provided in the Data Supplement).

### Previous Screening

The use of health services was statistically significantly higher in women who had a previous Pap smear or VIA. Characteristics of women with and without a history of screening are presented in Table 5, and characteristics of women categorized by use of health services are presented in the Data Supplement. The final adjusted model included age and education level, as well as the HPV test results. The participants’ use of alcohol, as well as other demographic factors, were considered but not included in the final model.

**Table 5:**
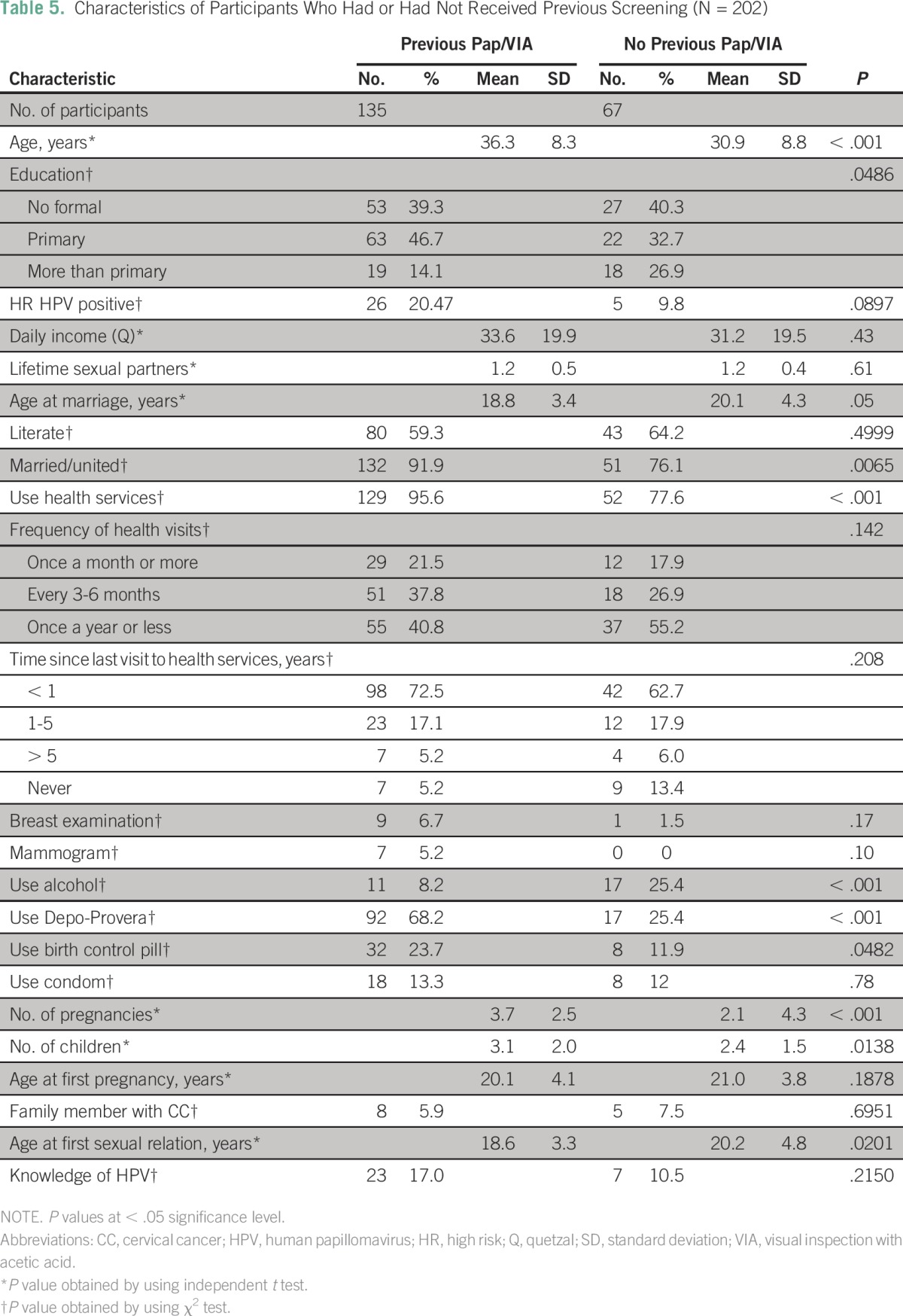
Characteristics of Participants Who Had or Had Not Received Previous Screening (N = 202)

After adjustment, the association between use of health services and having had a previous Pap smear or VIA remained greater than 1 but was no longer significant (crude PR, 2.49; 95% CI, 1.26 to 4.93; *P* = .009; adjusted PR, 1.24; 95% CI, 0.93 to 1.66; *P* = .15; regression tables are provided in the Data Supplement).

## DISCUSSION

We assessed the acceptability of HPV self-collection as an alternative to screening by using Pap smears or VIA in an indigenous community in Latin America. We found that self-collection kits had high acceptability and were preferred to physician screenings; a majority of women found the test kit comfortable and easy to use. We found a 17.4% prevalence of high-risk HPV, which is consistent with previous studies reporting a 16.1% prevalence for Latin America.^43^ We also investigated risk factors for HPV infection and previous Pap smears or VIAs, associations that became statistically nonsignificant after adjustment for other covariates. This could be the result of inconsistencies with self-reporting or perhaps because their partner’s sexual history (which was not assessed) might be a stronger determinant of HPV risk in this community.

This study was intended to serve as a first step to determine the potential of HPV screening in indigenous populations and also to provide baseline data for future longitudinal studies assessing the efficacy of HPV testing versus other screening modalities. Perhaps the most relevant finding is the high acceptability of self-collection and the willingness of the participants to engage in the study. In fact, 95% of participants completed the survey, 93% were interested in collecting self-samples, and more than 90% called to receive their results, numbers higher than expected. The study was very well received in the community, with strong support from local and health authorities, suggesting the potential to eventually implement HPV screening programs in this and other similar settings.

Strengths of the study include the multiclustered community design, which allowed us to obtain a representative sample of the population, provided an opportunity for participants to try self-collection in their homes rather than at a clinic, and allowed local CHWs to perform recruitment and interviews. Because of the latter, interviews were conducted in the participants’ native language, potentially making them more comfortable answering sensitive questions. In addition, data were collected electronically, which eliminated the risk of errors from manual data entry. However, there are also important limitations. Given the cross-sectional design, participants might have misreported their history of screening and other risk factors, especially if there had been community educational programs or interventions that suggested that women should be screened for CC. Women may not have accurately remembered whether they had previously had a Pap smear or VIA (recall bias) or may not even be aware of whether these procedures had been performed on them. Another limitation is that we were unable to assess whether HPV-positive women followed up on their results. This is the topic of our current work in multiple communities in Guatemala with a new study population that will be followed up after 6 months and 1 year.

In addition, this community has been exposed to prior health interventions and studies from multiple institutions.^44-46^ Although these studies did not specifically discuss HPV and CC, the exposure to health interventions could be reflected in the women’s knowledge of health issues and their willingness to try self-collection. In the future, it will be important to assess the acceptability of these tests in other indigenous communities with less exposure to studies and interventions.

The study results are consistent with those of previous studies conducted in Asia and Africa on the acceptability of self-screening for HPV.^25,27,29^ However, to the best of our knowledge, this is the first study to assess self-collection in indigenous populations in Latin America. This is also one of the first studies to provide an opportunity for participants to collect a sample in a community setting rather than simply sharing their feelings toward self-collection or collecting at a clinic.

This work assessed the acceptability of HPV self-screening in one community in Guatemala. Guatemala is a country with 23 languages and even more distinct communities, so our findings cannot be generalized to the whole population. It will be important to attempt to replicate the study in other parts of Guatemala and Latin America. Although it does seem that HPV self-collection screening could be a useful alternative to Pap smear or VIAs in these settings, this information alone does not allow us to make any determinations about whether this method of screening will reduce CC rates in developing countries. Women who tested positive for HPV should follow up with a doctor to receive Pap smears or VIAs or treatment. Hence, a logical next step would be to conduct longitudinal studies that compare rates of follow-up care among women who have tested positive with rates for those who have not been screened for HPV, as well as head-to-head comparisons between HPV-based versus Pap smear and VIA screening programs.^47^ It is also important to continue developing new affordable and easy-to-use tests that could readily be implemented in low-income settings.^20-22^

The Ministry of Health in Guatemala is in the process of refining the National Cervical Cancer Prevention and Control program.^48^ Following Pan-American Health Organization and WHO guidelines, the ministry has compiled a list of screening programs, some including HPV testing, that could be adopted. It will be the responsibility of each province (department) to determine which program best fits their needs and resources. We hope that our study, along with future evidence,^49^ will help local and regional authorities identify the best CC screening alternative for their own settings.
